# Assessment of Acute Pain Management and Associated Factors among Emergency Surgical Patients in Gondar University Specialized Hospital Emergency Department, Northwest Ethiopia, 2018: Institutional Based Cross-Sectional Study

**DOI:** 10.1155/2018/5636039

**Published:** 2018-12-02

**Authors:** Amare Agmas Andualem, Girmay Fitiwi Lema, Yonas Addisu Nigatu, Seid Adem Ahmed

**Affiliations:** ^1^Department of Anesthesiology, College of Medicine and Health Sciences, Wollo University, Dessie, Ethiopia; ^2^Department of Anesthesia, College of Medicine and Health Sciences, University of Gondar, Gondar, Ethiopia

## Abstract

**Background:**

Adequate pain management has led to increased comfort in emergency patients, reducing morbidity and improving long term outcomes. Different pain management modalities have been applied in the emergency department among which systemic analgesia is commonly used by preceding a nerve block. Several factors have been associated with poor pain management in low resource setting areas. We aimed to determine pain management modalities and associated factors among emergency surgical patients.

**Patients and Methods:**

After obtaining ethical approval from Ethical Review Committee, 203 volunteer patients were enrolled. Institutional based cross-sectional prospective study was conducted from April to May 2018 in Gondar University Specialized Hospital Emergency Department. The severity of pain was measured through Numerical Rating Scale and statistical analysis was performed using SPSS statistical package version 23. Descriptive statistics cross-tab and binary logistics were performed to identify factors related to pain management in emergency department.

**Results:**

A total of 203 patients, 138 (68%) males and 65 (32%) females with response rate of 94%, participated in this study. Among them, 66% patients received analgesia within two hours of ED presentation with a mean ± SD of 61.0 ± 34.1 minutes. 70.4 % of patients complained of moderate and severe pain after receiving analgesia. There was a significant difference between trauma and nontrauma patients in mean time of analgesia receiving and residual pain severity (p < 0.001). Age, trauma, physician pain assessment, and severity of pain were the predicting factors for analgesia delivery.

**Conclusion:**

The overall practice of pain management in Gondar University Specialized Hospital Emergency Department was not adequate. Therefore, it is vital to implement an objective pain assessment method and documentation of the pain severity to improve pain management practice.

## 1. Background

Pain is the main reason triggering patients to seek help in emergency department. International Association of Pain Study (IAPS) defines pain as an unpleasant sensory and emotional experience arising from actual or potential tissue damage. Pain affects all age groups without discriminating individuals based on gender or ethnicity [1].

Analgesia plays an important role in speeding the patient's recovery, reducing morbidity as well as improving clinical outcomes. The Emergency Department (ED) should be able to provide different services including pain management. Several studies have reported that pain is not treated adequately in the ED even though suitable analgesic drugs and different techniques are available. Moreover, several studies have identified different reasons for poor pain management in ED including an attitude of suspicion, a culture of ignoring the problem, and an environment that is not suitable for change in practice [2–8].

Lack of guidelines, regular training sessions, and measurement pain could play a role but these factors have not been widely studied [7]. Patient age, gender, disease patterns, and the relationship and communication between physicians, nurses, and patients seem to have an important impact on pain management [6, 7, 9, 10]. In addition, a systemic review done in 2009 has showed that failure to acknowledge pain, failure to assess initial pain, failure to have pain management guidelines in ED, failure to document pain, failure to assess treatment adequacy, and failure to meet patient's expectations are causes of poor pain management [11].

The prevalence of acute pain in the ED has been widely recognized and evidences support that 7 out of 10 patients come to ED because they are in pain [9]. In the United State of America from 2000 to 2010, approximately 45.4% of ED visits were associated with a primary symptom or diagnosis of pain. The proportion of pain visits as a patient-reported symptom or physician diagnosis remained stable and consistently represented approximately 45% of ED visits. Patients reported pain as their primary reason twice as often as providers reported a primary diagnosis of pain (~40% vs ~20%) [10]. In the USA and Canada multicenter research showed that 70 % of emergency patients suffer moderate and severe pain in ED but only 60% of patients receive analgesics [12]. A study done in Nigeria revealed that after analgesia delivery 85 % of patients in ED felt severe and moderate pain [13]. Another study done in Netherland showed that 91% of patients feel pain at admission and 86% on discharge [14]. Brown and his colleagues in 2000 showed that in ED analgesia for patients with isolated closed fractures of the extremities 64% received any type of analgesic and 42% received a narcotic analgesic [15].

In Switzerland a survey among emergency physicians and anesthesiologists involved in ED pain management responded that morphine is the most frequently used opioid in the ED (41%), followed by nicomorphine and pethidine. Furthermore, propacetamol and ketorolac are the most frequently used intravenous nonopioids in the emergency room reported by 26% of respondents [2].

A randomized controlled trial done by Mahshidfar and his colleagues in 2017 showed that 0.2mg/kg of ketamine has a significant reduction of acute pain when compared to that of 0.1mg/kg IV morphine ED [16].

A study done in USA on geriatric hip fracture patients showed that patients who received fascia iliac compartment block (6% vs 60% ) in the ED had a shorter time to first analgesic use (93 minutes VS 103 minutes) and received fewer morphine equivalents in the first 24 hours [17]. In addition, a study done by Beaudoin and his colleagues showed that there is a significant decrease in pain intensity with regional nerve blocks in ED and decreased amount of rescue analgesia [18].

Guidelines for the management of pain in the ED have been introduced by the British Association of Accident and Emergency Medicine. Patients with severe pain (pain score, 7-10) should receive appropriate analgesia within 20 minutes of arrival or triage. Patients with moderate pain should be offered analgesia at triage [5]. However, a study done in Singapore showed that the mean waiting time from arrival at the ED to the time the patient received analgesia was 77.6 minutes for only trauma patients. The median waiting time to analgesia was 70 minutes (minimum, 18 minutes, maximum, 243 minutes). The 25th and 75th percentile for time to analgesia were 47 minutes and 90.5 minutes, respectively [19].

A prospective cohort study done in Nigeria showed that 58% were trauma patients and 42% were nontrauma cases presenting with pain in ED. The mean pain score for all patients on the VAS was 6.9. The trauma cases had significantly lower VAS scores than the nontraumatized patients (6.1 vs 8.1). Analgesia was not prescribed in 45.2% of the patients, 65% of whom were with severe pain. In addition, 81% who were given preoperative analgesia had moderate to severe residual pain [13].

Agodirin and his colleagues performed a randomized controlled trial in Nigeria showing that preoperative use of tramadol does not affect the accuracy of the diagnosis of acute abdomen in ED [20]. In addition to this, several studies showed that preoperative use of opioids does not affect the accuracy of diagnosis of acute abdomen. Moreover, the use of analgesics in acute abdominal pain significantly improves patient comfort without compromising management decisions [21–24].

This study was designed to determine pain management modalities and associated factors among emergency surgical patients in Gondar University Specialized Hospital Emergency Department.

## 2. Methods

### 2.1. Study Setting and Population

After obtaining ethical approval from ethical review board of college of medicine and health science, an institutional based cross-sectional prospective study was conducted from April to May 2018 in Gonder University Specialized Hospital Emergency Department, located in Gondar town. Gondar is the capital of North-Gondar administrative zone in Amhara regional state, located 748 km northwest of Addis Ababa. The hospital is estimated to serve over 5 million people around the area.

All adult emergency surgical patients aged greater than 18 years who were present at the ED during the study period were enrolled in this study. Surgical patients having chronic pain, Glasgow Coma Scale < 14, and documented cognitive disability, uncooperative patients, and patients who had multiple site injuries and hemodynamically unstable patients were excluded from the study.

### 2.2. Sample Size and Sampling Procedure

The sample size was calculated using a single population proportion formula **n= (Z**_**α**/2)_^2^**p(1-p)/ω**^2^ where **n** is the minimum sample size, **Z** is the standardized normal distribution value at **α**/2**, P** is the incidence/proportion of pain, and* d* is the margin of error. A study done in Nigeria on preoperative analgesia in emergency surgical care showed that the incidence of pain is** 85**%** (p=0.85)** [20], **Z**_**α**/2_** at 95**%  **CI (1.96),** the sample size was 196, by adding 10% for nonresponse rate, and 216 participants were involved in the study. Consecutive sampling technique was used in this study.

### 2.3. Data Collection and Quality Assurance

Sociodemographic variables, causes of ED admission, physician's pain assessment, and pain documentation and management modalities were collected by using chart review and a pretested structured questionnaire. The questionnaire was designed and modified appropriately and translated into local language (Amharic) to be understood by all participants and translated back to English again to ensure its consistency. Training was given for four data collectors and one supervisor. Pretest was done on 15 emergency surgical patients two weeks before the day of actual data collection. The data collectors were supervised daily, and the filled questionnaires were checked by the supervisor and the principal investigator. Pretested respondents were not included in the main study.

### 2.4. Data Processing and Analysis Procedures

Sociodemographic variables, severity of pain, associated factors, and management modalities of pain data were entered and analyzed using SPSS 23.00 version statistical software. The severity of pain and management of pain were expressed as descriptive statistics and binary logistic regression was used to adjust or control the possible confounding factors and to identify associated factors of pain in the ED. The cut point for statistical significance was P < 0.05.

### 2.5. Ethical Considerations

Ethical clearance was obtained from Ethical Review Committee of College of Medicine and Health Science University of Gondar. Written informed consent was obtained from each participant after clear explanation of what they would have to do to take part in the study. Anyone not willing to participate in the study was informed that they had full right not to participate or withdraw at any time. Confidentiality was guaranteed by keeping the secrecy of personal identification, keeping completed questionnaires and results in well secured area.

## 3. Results

### 3.1. Sociodemographic Characteristics

A total of two hundred and sixteen emergency surgical patients were studied. Thirteen patients were excluded from analysis for incomplete data. Among the study participants 138 (68%) were males and 65 (32%) were females. Fifty-nine percent of the respondents were adults with range of 18-29 and the mean (±SD) age was 33.1±14.6 years (Table 1).

### 3.2. Causes of ED Admission

The main reason for ED presentation was trauma, 134 (66%), which resulted from domestic violence 48 (23.6%) followed by motor vehicle accident 44 (21.7%). Nontrauma ED presentation accounted for 34% of patients. Nontrauma patients came with appendicitis, large bowel obstruction, and small bowel obstruction which were relatively common causes of ED admission with 18(8.9%), 16(7.8%), and 12(5.9%) patients, respectively. Fracture and soft tissue injury were the two common injury types for trauma patients, 58 (28.6%) and 66 (32.5%), respectively (Table 2).

### 3.3. Analgesia Delivery in ED

During presentation in ED 25 (12.3%) patients with trauma received analgesia; however, nontrauma cases did not receive analgesia within 30 minutes of presentation. After two hours of ED presentation, there was a highly significant difference in analgesia given between trauma and nontrauma with a p value < 0.001. Among nontrauma cases only 34 (49.3%) of patients received analgesic drugs but among trauma patients 100 (74.6%) of patients received analgesic drugs (Table 3).

The mean time to first analgesia was 60.98 + 34.05 minutes for all emergency surgical patients. The mean time of analgesia was also statistically significant between trauma and nontrauma patients with a p value < 0.001 (Table 3).

### 3.4. Severity of Pain

Pain severity during presentation at ED and two hours after presentation was 7.67±1.89 and 5.19 ±2.35, respectively, and there was a significant difference between trauma and nontrauma patients both at ED presentation and at two hours after ED presentation with a p value 0.016 and < 0.001, respectively, but there was no statically significant difference between males and females on pain severity (Table 4).

**Figure 1:**
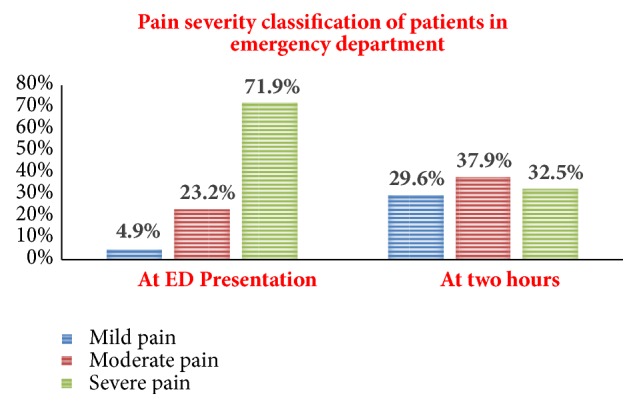
Classification of pain severity at presentation after two hours in GUSHED, Northwest Ethiopia, 2018 (N=203).

The severity of pain in ED was expressed according to WHO classification after NRS score obtained. At ED presentation 193 (95.1%) emergency surgical patients presented with severe and moderate pain. After two hours of ED presentation 32.5%, 37.9%, and 29.6% emergency surgical patients complained from severe, moderate, and mild pain, respectively (Figure 1).

### 3.5. Type of Analgesia Used at ED

**Figure 2:**
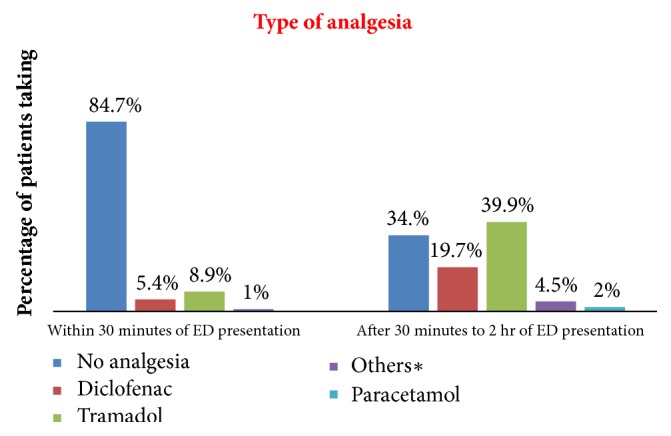
Specific analgesic medication usage in GUSHED, 2018 (N= 203). ^∗^Strong opioids and local anesthetics.

Tramadol was given to 81 (39.9%) of patients followed by diclofenac 40 (19.7%) at 2 hours after ED presentation. 69 (34%) of patients did not receive any analgesia in ED. Among those who did not receive analgesia 61 (88.4%) of patients reported analgesic medications were not prescribed. Overall 107 (57 %) patients reported that the analgesic was not adequate and needed additional analgesia (Figure 2).

### 3.6. Associated Factors of Pain Management at ED

Factors associated with analgesia delivery in ED were age of the patient, cause of ED admission, physician pain assessment, and severity of pain. Patients aged 30-59 were 4 times more likely to receive analgesia compared to those greater than 60 years (p = 0.024). Trauma patients were also four times more likely to receive analgesia than nontrauma patients (p < 0.001). Pain assessed by physicians was an independent factor but only 41(20.2%) of patients were assessed. Patients assessed by a physician were three times more likely to receive analgesia than those not assessed (p=0.02). A patient presenting at ED with severe pain was 3.5 times more likely to receive analgesia compared to those with mild pain (p= 0.007). Even though patient analgesic request was not associated with analgesic administration in the ED, 128 (63.1%) of patients requested analgesia during ED stay and 75 (36.9%) patients did not request analgesia. The main reasons for not requesting analgesia were that patients, thought the physicians did what was important for them, hoped the pain would get better, had no idea about analgesia, thought asking was culturally unethical, and lastly had fear of drug complications 42(56%), 16(21.3%), 14(18.7%), 2(2.7%), and 1 (1.3%), respectively (Table 5).

## 4. Discussion

This observational study was conducted to determine the practice of pain management modalities and associated factors among emergency surgical patients in ED. The mean severity of pain during ED presentation was 7.67 ± 1.89, which was a high mean of pain severity when compared to a study in Nigeria which was 6.9 ± 2.5 [13]. With this mean value of pain severity, according to WHO pain severity classification 71.9%, 23.2%, and 4.9% of patients complained of severe, moderate, and mild pain during ED presentation, respectively. In keeping with other studies the incidence of acute pain ED was significantly high when expressed as 95.1% (moderate and severe pain) of patients in pain; this result was higher than that of a study by Berben and his colleagues in Netherland which was 91% during ED admission. In this study however they include only trauma patients and it is likely that patients present early to the ED possibly with milder pain [14].

Residual pain after two hours of ED presentation was 5.19 ± 2.35. Similarly, in Pakistan, a study showed that postanalgesic mean residual pain score of all patients was 5.0±1.8. This score was less in trauma patients (4.7±1.9) as compared to acute abdomen patients (5.7±0.9). In this study mean residual pain for nontrauma was higher which could be explained by a delay to administer analgesia to these patients, specially for acute abdomen because of fear of masking the signs for correct diagnosis [25]. By classification of severity of residual pain after two hours of ED presentation 29.6%, 37.9%, and 32.5 % of patients complained of mild, moderate, and severe pain, respectively. In contrast to our study, the study in Nigeria showed that 85% of patients had moderate and severe pain and 15% of patients had no to mild pain after analgesia but it included only acute abdomen patients which may result in high scores compared to our result [13].

In this study only 12.3% of patients received analgesia within 30 minutes of ED presentation in contrast to the British Association of Accident and Emergency Medicine (BAEM) guidelines. These state that patients with severe pain should receive appropriate analgesia within 20 minutes of arrival and those with moderate pain should be offered analgesia at triage [5]. Within 2-hour duration only 134 (66%) of our patients received analgesia and 69 (34%) patients did not receive analgesia. In this study there were fewer analgesics given for nontrauma patients possibly due to the withholding of analgesia for acute abdomen until definitive surgical management. In Nigeria no preoperative analgesia was prescribed for 45.2% of patients, the majority of whom had moderate or severe pain [13]. In Netherlands only 83 (19%) of patients received pharmacological pain treatment which showed that lack of analgesia is a problem in both developing and developed countries [14].

The mean time to delivery of analgesia was 61 ± 34 minutes, three times longer than the British Association of Accident and Emergency Medicine acute pain guidelines. In a study in the UK the mean time of arrival to analgesia was 72 minutes for severe pain and 236 minutes for moderate pain [5]. In contrast to this study, a study from New York showed that the mean time to analgesia for Emergency Medical service (EMS) treated patients was 23 minutes and mean time to analgesia after triage in this group was 75 minutes. This included the use of analgesics given at the scene of injury before triage [26]. A study done in Singapore showed that the mean waiting time from arrival at the ED to the time the patient received analgesia was 77.6 minutes. The median waiting time to analgesia was 70.0 min (minimum, 18.0 min; maximum, 243.0 min). The 25th and 75th percentile for time to analgesia were 47.0 min and 90.5 min, respectively [19]. However, in our study medical interns initiate analgesia in the ED which may delay analgesia and give emphasis for diagnosis. Two studies demonstrate that a nurse initiated analgesia system can shorten the time to receive analgesia in ED [27, 28].

The type of analgesia used in ED varies from hospital to hospital and from country to country. In this study 39.9% of patients received tramadol and 19.7% received diclofenac. The majority (95.1%) of patients complained of moderate and severe pain in the ED. Similarly a study from Jimma, Ethiopia, showed that tramadol was also the most prescribed analgesic for postoperative pain management [29]. This may be due to the difficulty accessing opioids in the study area. In addition, a study done by Wosenyeleh and his colleagues in Gondar showed that diclofenac 40 (27%) and pethidine 11 (7%) were the most commonly given intraoperative analgesic drugs for both emergency and elective surgical procedures done which shows a limited usage of potent opioids in the study area [30].

In contrast to this study, a study by Todd and his colleagues showed that the majority of analgesics administered were opioids (59%). Morphine was the single most commonly administered analgesic (20%), followed by ibuprofen (17%) [6, 31].

Berben and his colleagues showed that 33 (40%) of ED pain treatment consisted of local anesthesia, for suturing or repositioning of fractures. The other 51 (60%) received systemic pain medication, explained as they included patients for discharge including procedural analgesia [14]. Nerve block can be effective for trauma patients at ED depending on the site of injury. A study done by Mahshidfar and his colleagues showed that low dose of ketamine (0.2mg/kg) resulted in a significant reduction of pain when compared to that of intravenous morphine with fewer complications than morphine in early presentation of patients in ED [16]. Ketamine was not used out of operation theatre in the study area, however, which is an alternative for developing countries with a low accessibility for potent opioids.

There are many different factors which result in undertreatment of pain in the ED [32]. In this study, age, cause of admission, severity of pain, and physician pain assessment were the independent risk factors. A patient presenting with nontrauma had a risk of inadequate analgesia or did not receive analgesia with an AOR of 3.99 (95% CI; 2.01-7.94) compared to a trauma patient. This was explained by emergency surgical care for acute abdomen avoiding analgesia until confirmed diagnosis and management. However, reviews, RCTs, and guidelines recommended adequate analgesia for acute abdomen after the presentation in the ED. Moreover, the use of analgesics in acute abdominal pain significantly improves patient comfort without compromising management decisions [20–22, 24, 33].

Age greater than 60 was associated with oligoanalgesia. Adult patients within the age range of 30 to 60 received analgesia four times as often (95% CI 1.14-13.65) as those with an age greater than 60. In a study similar to this Jones and his colleagues showed that 66% of elderly patients received analgesia compared to 80% of their younger counterparts. They also had significant underdosing of pain medication and received less opioid analgesia [34]. A meta-analysis in 2017 supports this idea by explaining there was an increment of pain threshold in geriatrics patients. Elderly patients may have atypical manifestations of pain and healthcare providers avoid potent analgesics due to concern over the respiratory depressant effects of opioids because of alteration of pharmacokinetic and pharmacodynamics changes [11, 35].

Physician pain assessment was the one predictor to getting analgesia at ED. In this study pain assessment was performed in only one-fifth (20.2 %) of patients and was significantly associated with analgesia delivery. A study by Silka and his colleagues showed that pain scoring in the ED improves analgesia administration for trauma patients [36].

Severe pain was associated with analgesia delivery compared to mild pain with AOD ratio of 3.09 (95% CI; 1.42-8.54). In a similar study to this in Central Africa by Rampanjato and his colleagues pain severity was highly associated with the median time of analgesia administration by nurses [37]. After assessment of pain, documentation was also one factor for analgesia delivery in ED. In this study there was inadequate pain documentation and reassessment of pain. A study by L. Sturesson and his colleagues showed that the percentages of patients receiving analgesic drugs increased and pain intensity decreases at discharge were statistically significant after an intervention that made nurses obliged to register pain in ED [38].

## 5. Limitation of the Study

This study focused on the clinical perspective of pain management modalities and did not look at knowledge and attitudes of emergency healthcare providers and patients which are confounding factors for poor pain management. The physician to patient ratio was not analyzed which has also been identified as a factor in previous studies for poor pain management in ED.

## 6. Conclusion and Recommendations

The practice of pain management in the ED was inadequate. The type of analgesics administered did not match the severity of pain. The time to analgesia was delayed and one-third of emergency surgical patients did not receive analgesia within two hours of ED presentation. Even though the severity of pain decreased after two hours of ED presentation, the prevalence of pain after treatment was high. Moreover, lack of objective pain assessment, lack of documentation of the severity of pain, and time of analgesia at GUSH surgical ED were the independent risk factors for poor pain management.

According to the result of this research we recommend that the healthcare staff should administer adequate analgesics to the nontrauma patient in equal measure to trauma patients. In addition, healthcare providers should assess and document severity of pain as a fifth vital sign so as to administer appropriate analgesic drugs including potent opioids and regional nerve blocks.

Furthermore we recommend further study on knowledge and attitudes of emergency department health workers towards pain management.

## Figures and Tables

**Table 1:** Sociodemographic characteristics of participants at GUSHED, 2018 (N=203).

**Variables**		**Numbers**	**Percent (**%**)**
**Sex **	Male	138	68
	Female	65	32
**Age **	18-29	120	59.1
	30-59	63	31.0
	>60	20	9.9
**Ethnicity **	Amhara	191	94.1
	Tigray	12	5.9
**Residency **	Urban	108	53.2
	Rural	95	46.8
**Level of education **	Can't read and write	41	20.2
	Can read and write only	33	16.3
	Primary education	36	17.7
	Secondary education	48	23.6
	College and above	45	22.2

**Table 2:** Causes of emergency department presentation at GUSHED, 2018 (N=203).

**Variables**			**Frequency (n)**	**Percent (**%**)**
**Causes of admission**	**Trauma**	Fracture	58	28.6
		Dislocation/ sublaxation	16	7.9
		Soft tissue injury	66	32.5
		Superficial lacerations	11	5.5
		Chest injury	5	2.5
		Wound	8	3.9
		Others^∗^	8	3.9
		Subtotal	172^∗∗^	84.7^∗∗^
	**Non trauma**	Appendicitis	18	8.9
		Small bowel obstruction	12	5.9
		Large bowel obstruction	16	7.8
		Breast abscess	6	3.0
		Perianal abscess	6	3.0
		Others^∗∗∗^	11	5.4
		Subtotal	69	34

^*∗*^Pelvic injury, blunt abdominal injury, eye injury, and ear injury.

^∗∗^38 trauma patients came with two types of injuries.

^∗∗∗^Cellulitis, gastric outlet obstruction, nephrolithiasis, urinary stricture, and axillary abscess.

**Table 3:** Analgesia and timing of analgesia among participants in GUSHE, Northwest Ethiopia, 2018 (N=203).

**Characteristics **		**Trauma (**%**)**	**Non trauma (**%**)**	**p-value**
**Analgesia received **	**Yes**	100(49.3)	34(16.7)	< 0.001^∗^
	**No**	34(16.7)	35(17.2)	
	**Total**	134(66)	69(34)	
**Timing of analgesia ( mean ±SD)**		53.2 ± 30.1	88.8 ± 33.1	< 0.001^∗∗^

^*∗*^Chi-square was used to analyze significant difference between two groups. P value less than 0.05 was statistically significant.

^∗∗^Independent sample t-test was used to compare the mean time of analgesia between trauma and nontrauma. P value less than 0.05 was significantly significant. SD= standard deviation.

**Table 4:** Pain severity using NRS among emergency surgical patients in GUSHED, Northwest Ethiopia, 2018 (N=203).

**Participants**	**Pain severity at presentation of ED**	**p-value**	**Pain severity at two hours after admission**	**p-value**
**All (n=203) **	7.67 **±**1.89	-	5.19 + 2.35	-
**Male ( n=138)**	7.57 ± 1.95	p = 0.28	4.92 ± 2.28	p = 0.16
**Female (n=65)**	7.87 ± 1.77		5.76 ± 2.34	
**Trauma (n=69)**	7.45 ± 2.00	p = 0.016	4.67 ± 2.20	p < 0.001
**Non trauma (n=134)**	8.09±1.59		6.20 ± 2.33	

Data are presented as mean ± SD, Independent sample t test was used to compare the mean pain severity between trauma and non-trauma. p-Value less than 0.05 was significantly significant.

**Table 5:** Associated factors for analgesia delivery in GUSHED, Northwest Ethiopia, 2018 (N= 203).

**Factors**	**Group**	**P value**	**Odds ratio**		**95**%** CI**	
			**COR**	**AOR**	**Lower**	**Upper**
**Age **	18-29(59.1%)	0.379	1.23	1.61	0.56	4.64
	30-59(31.0%)	0.024	2.40	4.04	1.14	13.65
	≥60(9.9%)	0.035				
**Cause of ED admission**	Non-trauma (34%) Trauma (66%)	<0.001	3.02	3.99	2.01	7.94
**Physician pain assessment**	No (79.8%) Yes (20.2%)	0.02	3.71	3.09	1.18	8.06
**Pain severity at presentation**	Mild (4.9%)	0.017				
	Moderate (23.3%)	0.630	0.62	0.71	0.18	2.83
	Sever (71.9%)	0.007	3.03	3.49	1.42	8.54
**Patients analgesic request**	No (36.9%) Yes (63.1%)	0.168	1.52	-		

*Multivariate logistic regression analysis was done. P value less than 0.05 was statistically significant.*

*AOR=adjusted odds ratio, COD=crude odds ratio, and CI= confidence interval*.

## Data Availability

The data used to support the findings of this study are available from the corresponding author upon request.

## Abbreviations

APS: American Pain Society
ED: Emergency Department
FNB: Femoral nerve block
GCS: Glasgow Coma Scale
GUSH: Gondar University Specialized Hospital
IAPS: International Association of Study of Pain
Kg: Kilogram
Mg: Milligram
NRS: Numerical Rating Scale
OR: Odds ratio
RCT: Randomized controlled trial
VAS: Visual analogue scale
WHO: World Health Organization.
